# Making Patients Pay: Informal Patient Payments in Central and Eastern European Countries

**DOI:** 10.3389/fpubh.2015.00192

**Published:** 2015-08-07

**Authors:** Tetiana Stepurko, Milena Pavlova, Irena Gryga, Wim Groot

**Affiliations:** ^1^School of Public Health, National University of Kyiv-Mohyla Academy, Kiev, Ukraine; ^2^Department of Health Services Research, CAPHRI School for Public Health and Primary Care, Maastricht University Medical Center, Faculty of Health, Medicine and Life Sciences, Maastricht University, Maastricht, Netherlands; ^3^Topinstitute Evidence-Based Education Research (TIER), Maastricht University, Maastricht, Netherlands

**Keywords:** informal patient payments, corruption in health care, patient charges, attitudes, perceptions

Informal patient payments are a key characteristic of nearly all Central and Eastern European health care systems (1–3). Apart from formal patient payments, which are regulated by national legislation (4) and quasi-formal charges, which are set by the health care provider in the absence of clear government regulations (5–7), there are also informal payments (also known as “under-the-table” or “envelope” payments), which comprise the unregistered patient payments for publicly funded health care services (7–9). In addition to this, there are also quasi-informal payments for goods that should be provided free of charge to the patient by the health care establishment but that patients are asked to purchase outside and bring for the treatment. Indeed, out-of-pocket patient payments are a major source of health care funding in Central and Eastern European countries (10).

Informal patient payments warrant special attention as ignoring these payments causes an underestimation of total health expenditure and their hidden nature imposes a great challenge to the health care provision in terms of accessibility as well as accountability and transparency (2, 11–13). Informal payments constitute about 1.5–4.6% of total expenditure on health in Hungary, about 0.3–0.5% in Poland, and about 2% in Bulgaria (14). Furthermore, a few decades ago, informal patient payments were considered mostly as “gratitude money,” or a socio-cultural phenomenon (5). Currently, multi-dimensional explanations, such as insufficient resources (low income of physicians) and inadequate governance (poor political-regulatory context) combined with the socio-cultural reasons prevail in the literature (15–17). These three dimensions are rather interwoven leading jointly to the existence of a specific pattern of informal patient payments in a country.

Empirical studies on informal payments are one of the main sources of evidences on this multi-faceted phenomenon; however, they comprise a variety of methodological challenges (7), including (a) the identification of a suitable sample unit (patients, citizens, providers, and/or officials), (b) a socio-culturally acceptable data collection mode (face-to face interviews or self-administrated questionnaires) (18, 19), and (c) adequate operational definitions of informal patient payments because some respondents find it difficult to distinguish between formal and informal payments (9, 20). The difficulties related to an adequate methodology design and implementation may explain the focus of most empirical studies on single countries and on the scale and determinants of these payments rather than on complex multi-country comparative studies (7). Still, a huge variety in the nature and patterns of informal patient payments is reported across countries (7). Studies provide evidence on the variation in the type of informal payments (cash or in-kind gifts given by patients or their families), timing (before, after or during service provision), subject (out- or in-patient service), purpose (obtaining better quality or access), and motivation (physician’s request or patient’s initiative) (1, 3, 8).

Last but not least, the key characteristics of informal patient payments studied should also include the perceptions and attitudes toward these payments, which are the most indicative in a cross-country comparison. Indeed, evidence on perceptions and attitudes toward informal patient payments may play an essential role in developing and implementing adequate strategies for dealing with these payments (21–23). A cross-country comparison of public attitudes, perceptions, and opinions on informal patient payments is presented in Stepurko et al. (3). In 2010, representatives of households in Bulgaria, Hungary, Lithuania, Poland, Romania, and Ukraine consistently reported negative attitudes toward informal cash payments. This suggests a prevalence of corruption connotation, which is evidence of its social undesirability. In-kind gifts are more often perceived as gratitude than informal cash payments. More positive perceptions of informal payments in general are observed among those who have ever given in-kind gifts rather than those who have ever paid informally in cash. Concerning cross-country patterns, public perceptions in some countries (especially in Poland but also in Bulgaria) are less positive about informal patient payments than in other countries (specifically in Hungary and Ukraine). In terms of policy analysis and research, it is important to distinguish truly gratitude informal payments that have practically no impact on health care service provision, from other types of informal payments (bribes) that undermine the functioning of the health care system, and attitude study provides one piece to this puzzle.

Moreover, when perceived behavior related to making informal patient payments has been studied (24), it also appears that health care users in Bulgaria and Poland are less inclined to make informal payments, while health care users in Romania and Ukraine most often report such payments. The informal payment rates for Hungary and Lithuania fall between these two groups. In all six countries, individuals who feel uncomfortable when leaving the physician’s office without a gratuity and who feel unable to refuse the request of medical staff to pay informally, more often make informal payments. Additionally, it has been found that socio-demographic characteristics have lower relevance compared to perceived behavior. Indeed, the behavioral pattern of making informal patient payments is mostly associated with patient’s perceptions, while socio-demographic features play a minor role in explaining this pattern. Specifically, those who feel uncomfortable to leave without a gratitude payment and who feel unable to refuse to pay informally if asked, more often report making informal payments than the rest of the respondents. The less positive attitudes and perceptions toward informal patient payments in Poland can be attributed to the successful anti-corruption policies supported by mass-media and relatively better governance in the country than in neighboring countries (3).

Public opinions and individual perceptions provide a good ground for better understanding the level and patterns of informal patient payments. Variation in informal patient payments with regards to the country and year of occurrence, type of service used, the purpose and initiator of the payment shed more light on the roots of the informal payments, and therefore strategies for their eradication (25). The results of the cross-country comparison mentioned above suggest a relatively higher prevalence of informal patient payments in Ukraine than in Bulgaria, where patients also meet formal service charges in the public sector. More than 35% of health care users in Ukraine report informal payments for physician visits during the preceding 12 months in addition to widespread quasi-formal payments (26), while in Bulgaria it is <10%. Regarding hospitalizations, the percentage of service users who report informal payments is also higher in Ukraine (more than 40%) and lower in Bulgaria (10–20%). It should be noted that in Bulgaria, the private health care sector has considerably grown during the last decades, which provides alternatives to the public health care services. This can explain to a certain extent the lower share of informal patient payments in Bulgaria as Bulgarian patients may opt for private services and avoid informal payments in the public sector.

Informal payments are more spread and higher when they are solicited or expected by providers (25). However, the relatively high prevalence of informal patient payments in Hungary does not follow this logic since informal payments in Hungary are mostly initiated by the consumers (3). This underlines the importance of country-specific strategies for dealing with informal patient payments. In Hungary, in particular, the initial objective of such strategy should be the revision of national regulations, which at this moment are supportive to informal payments (23).

Furthermore, the probability and the size of the informal payment are to a great extent determined by the type of service consumed (GP or specialist, out-patient or in-patient care) (15, 27). The trend of a higher number of users who make more expensive informal payments to specialists when compared to GPs remains noticeable. It is similar for surgery and childbirth compared to other hospital interventions. Indeed, the number of payers and the amounts paid (including informal payers) are highest for hospitalizations related to childbirth or pregnancy (25). For example, in Ukraine, about half of the in-patients report informal payments for pregnancy or delivery, although the median value of these payments is about 70–100€ while total payment is about two to three times higher.

Insufficient data on maternity care provision in Eastern European countries have drawn the attention of researchers to the qualitative aspects of the process of informal payments (28, 29). Qualitative study conducted in the capital of Ukraine shed some light on the experience of consumers and providers with informal payment for childbirth (28). The methodology of ethnographic study has enabled us to learn more about local specificity of human behavior related to the process and nature of informal patient payments for childbirth. In 2008–2009, two groups of patients in the Ukrainian maternity care ward are identified: “individual patients” and “emergency room patients.” Also, push factors that lead to a search for a “personal obstetrician” in Ukraine are described as the need for 24 hours access to reliable information and the need for psychological comfort during the childbirth. Thus, gaining better “service wrapping” (reliable information, better attention, responsiveness) against the background of feelings of anxiety is seen by patients as a strategy to avoid “substandard care.” The obstetricians do not conceal their experience on the redistribution of the informal payments among medical staff as well as the use of money to buy pharmaceuticals and to maintain physicians’ wards. Informal payments not only add to the salary of the obstetricians but also to the salary of other staff members and to the budget of the hospital facility. In fact, the low salary of medical staff is indicated by both obstetricians and mothers as the main cause for the existence of informal payments. Thus, informal payments remain an unregulated tool that ensures extra payments to health care providers when adequate reimbursement policies are lacking.

Variations in regulatory mechanisms, availability of alternatives to informal payments as a means for achieving better quality and access, level and sources of funding can explain the cross-country diversity (30). Meanwhile, the devotion to accepting, giving, and relying on informal patient payments observed in the region can become a great obstacle in introducing health care reforms in any of the countries. Informal patient payments affect the health care system at the macro (system) level as they impede health care reforms, and at the micro (service) level by creating barriers to adequate care (1, 30). Most important, however, informal patient payments distort equity since patients who cannot afford to pay informally might be deprived from adequate health care (31). Thus, strategies for dealing with informal patient payments and their causes are urging.

However, the ability of the government to ensure a good performance of the public sector in general and of the health care sector, in particular, is seen as a key factor for avoiding shadow practices. So far, political decision-making in Eastern Europe has been too much based on the interests of business (the medical elites in case of health care) with too little consideration of evidence-based strategies and public opinion. This impedes real positive changes in public service provision regardless of the policy goals stated by the governments. Therefore, changes in governments and international organization interventions may give a stimulus to improve governance and the culture of management of all public sectors. The ultimate challenge for policy-makers is to realize that when informal patient payments appear in health care, it also aggravates the health and wealth of the nation.

## Conflict of Interest Statement

The authors declare that the research was conducted in the absence of any commercial or financial relationships that could be construed as a potential conflict of interest.
